# Acute Coronary Syndrome in the COVID-19 Era—Differences and Dilemmas Compared to the Pre-COVID-19 Era

**DOI:** 10.3390/jcm11113024

**Published:** 2022-05-27

**Authors:** Ratko Lasica, Lazar Djukanovic, Igor Mrdovic, Lidija Savic, Arsen Ristic, Marija Zdravkovic, Dragan Simic, Gordana Krljanac, Dejana Popovic, Dejan Simeunovic, Dubravka Rajic, Milika Asanin

**Affiliations:** 1 Department of Cardiology, Emergency Center, Clinical Center of Serbia, 11000 Belgrade, Serbia; lazardjukanovic08@gmail.com (L.D.); igormrd@gmail.com (I.M.); lidijasavic2007@gmail.com (L.S.); gkrljanac@yahoo.com (G.K.); dubravkarajic7@gmail.com (D.R.); masanin2013@gmail.com (M.A.); 2 Department of Cardiology, Clinical Center of Serbia, 11000 Belgrade, Serbia; arsen.ristic@med.bg.ac.rs (A.R.); dvsimic@yahoo.com (D.S.); dejanapopovic@yahoo.co.uk (D.P.); dejan.simeunovic@mfub.bg.ac.rs (D.S.); 3 Clinical Center Bezanijska Kosa, 11000 Belgrade, Serbia; sekcija.kardioloska@gmail.com

**Keywords:** acute coronary syndrome, COVID-19, myocardial injury, dilemmas in COVID-19, before COVID and COVID era

## Abstract

The COVID-19 pandemic has led to numerous negative implications for all aspects of society. Although COVID-19 is a predominant lung disease, in 10–30% of cases, it is associated with cardiovascular disease (CVD). The presence of myocardial injury in COVID-19 patients occurs with a frequency between 7–36%. There is growing evidence of the incidence of acute coronary syndrome (ACS) in COVID-19, both due to coronary artery thrombosis and insufficient oxygen supply to the myocardium in conditions of an increased need. The diagnosis and treatment of patients with COVID-19 and acute myocardial infarction (AMI) is a major challenge for physicians. Often the presence of mixed symptoms, due to the combined presence of COVID-19 and ACS, as well as possible other diseases, nonspecific changes in the electrocardiogram (ECG), and often elevated serum troponin (cTn), create dilemmas in diagnosing ACS in COVID-19. Given the often-high ischemic risk, as well as the risk of bleeding, in these patients and analyzing the benefit/risk ratio, the treatment of patients with AMI and COVID-19 is often associated with dilemmas and difficult decisions. Due to delays in the application of the therapeutic regimen, complications of AMI are more common, and the mortality rate is higher.

## 1. Introduction

The coronavirus disease of 2019 (COVID-19) is caused by severe acute respiratory syndrome coronavirus 2 (SARS-CoV-2). According to the World Health Organization, since the first case was registered in December 2019, about 476,000,000 people have been infected with the corona infection, while about 6,000,000 have died as a result [1]. Although predominantly a lung disease, COVID-19 exhibits many characteristics of systemic disease and has a major impact on the cardiovascular (CV) system. The most common risk factors that correlate with a more severe clinical picture and increased mortality from COVID-19 are the age of the patients, male gender, diabetes mellitus, presence of previous CVD, hypertension, chronic renal disease, and chronic obstructive pulmonary disease [2,3,4,5,6]. Patients with COVID-19 infection have associated CVD in 10–30% of cases [7,8]. Older age, obesity, and diabetes mellitus are not the only predictors of risk for the severity of the COVID-19 infection but are also previously known as strong predictors of CVD development in the general population. The interaction between SARS-CoV-2 and CV systems is two-way. While patients with pre-existing CVD and COVID-19 have poorer outcomes, the virus itself can cause heart injury and potentiate the occurrence of CV complications.

## 2. The Role of CVD in the Course of COVID-19 Infection

Patients with CVD and COVID-19 have a four-fold higher risk of death, compared to the COVID-19 patients in whom CVD is not present [9]. According to the results of the Docherty A.B. et al. study, 20,133 COVID-19 hospitalized patients showed that the presence of chronic CVD in patients with COVID-19 significantly increased the risk of mortality (HR-1.16, 95% CI 1.08–1.24, *p* < 0.001) [7]. The study by Fried M.W. et al. assessed the outcome and characteristics of patients hospitalized by COVID-19 across the United States. Of the total number of patients (11,721 patients), 18.6% had CVD and a statistically more frequent need for mechanical ventilation. Significantly higher mortality was registered in the group of patients with CVD than in the group of patients without CVD [8]. That CVD is significantly associated with a more severe clinical picture and poorer outcome in patients with COVID-19 was also confirmed by the results of a meta-analysis by Mishra P. et al. (OR 2.89; 95% CI 1.98–4.21 for severity and OR 3.00, 95% CI 1.67–5.39 for mortality, respectively) [10].

However, most of these studies did not differentiate between CVD types, but all CVDs were analyzed together, in correlation with severity and mortality from COVID-19 infection. Analysis of the CAPACITY-COVID registry and LEOSS study also showed higher mortality of patients with associated CVD in COVID-19, compared to those without CV comorbidity (29.7%; *n* = 1545 vs. 15.9%; *n* = 1797, respectively) [11].

## 3. Epidemiology and Etiology of ACS in COVID-19

**Figure 1:**
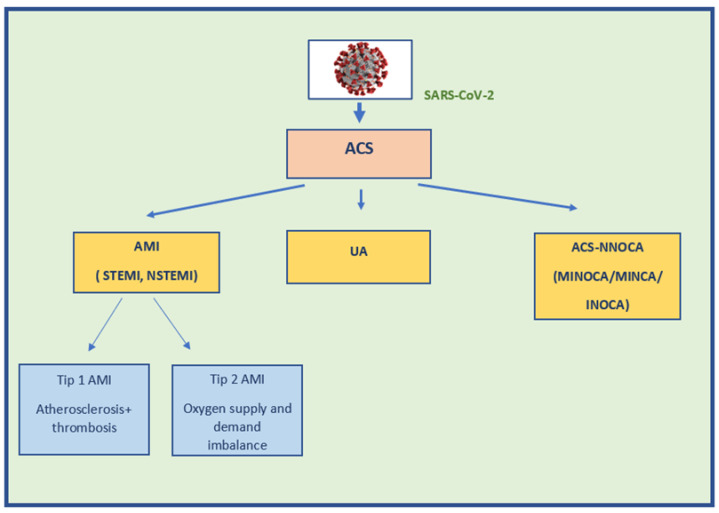
Different types of ACS in COVID-19. Legend: SARS-CoV-2—severe acute respiratory syndrome coronavirus 2; ACS—acute coronary syndrome; UA—unstable angina; AMI—acute myocardial infarction; ACS-NNOCA—acute coronary syndrome with normal or near-normal coronary arteries; INOCA—ischemia with non-obstructive coronary artery disease; MINCA—myocardial infarction with normal coronary arteries; MINOCA—myocardial infarction with non-obstructive coronary artery disease; NSTEMI—non-ST elevation myocardial infarction; STEMI—ST elevation myocardial infarction.

According to the “Fourth Universal Definition of Myocardial Infarction” from 2018, myocardial injury is defined as an increase in cTn value above the 99th percentile of the upper reference value (ULN). Myocardial injury is considered acute if there is a rise and/or fall of cTn values [12]. The term ACS refers to any group of clinical symptoms compatible with acute myocardial ischemia and includes unstable angina (UA), non-ST-segment elevation myocardial infarction (NSTEMI), and ST-segment elevation myocardial infarction (STEMI) [13]. The term AMI should be used when there is acute myocardial injury with clinical evidence of acute myocardial ischaemia and the detection of a rise and/or fall of cTn values, with at least one value above the 99th percentile URL and at least one of the following: symptoms of myocardial ischaemia, new ischaemic ECG changes, development of pathological Q waves, imaging evidence of new loss of viable myocardium or new regional wall motion abnormality in a pattern consistent with an ischaemica etiology, or the identification of a coronary thrombus by angiography or autopsy [12]. There is a group of patients with ACS without angiographic obstructive CAD (stenosis diameter ≥ 50% in the great epicardial vessel), and the term myocardial infarction with non-obstructive coronary arteries (MINOCA) has been coined for this entity [12] (Figure 1).

Acute heart muscle injury is common in patients with COVID-19 and correlates with disease severity [5,14]. Studies have shown that 7–36% of patients with COVID-19 infection develop myocardial injury [15,16,17]. The results of an observational multicenter cohort study (performed on 3108 COVID-19 patients) also showed an increase in the incidence of ACS in the COVID-19 era, compared to before the COVID-19 era (3.31% in 2020 and 1.01% in 2019) (*p* < 0.013) [18]. Katsoularis I. et al. suggested that the results of their study (86,742 patients with COVID-19 in the self-controlled case series and 348 481 matched control individuals in the matched cohort study) indicate that COVID-19 is a risk factor for AMI and ischemic stroke. Their data indicates that the risk of developing AMI in COVID-19 increased three to eight times, and the risk of developing ischemic stroke by three to seven times, with the highest incidence during the first two weeks after infection [19].

Patients with chronic coronary heart disease have an increased risk of myocardial injury, compared to patients who have not previously been diagnosed with coronary heart disease (43.8% vs. 14.4%) [20]. The Danish National Registry indicates that the incidence of AMI is five times higher within the first 14 days after being diagnosed with COVID-19 infection, compared to the period before infection [21]. The weighted pooled prevalence of ACS in patients with COVID-19 is 20% (range 5–38%) [22].

## 4. Pathogenesis of ACS in COVID-19

**Figure 2:**
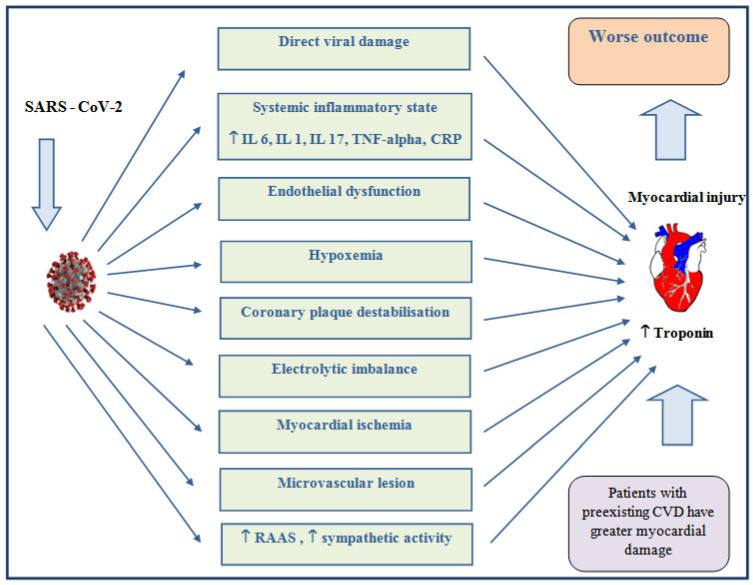
Possible mechanisms of myocardial injury in patients with COVID-19. Legend: IL 6—interleukin 6; IL 1—interleukin 1; IL 17—interleukin 17; TNF-alpha—tumor necrosis factor-alpha; CRP—C-reactive protein; RAAS—renin–angiotensin–aldosterone system; CVD—cardiovascular disease.

One of the mechanisms by which COVID-19 causes myocardial injury is the direct entry of SARS-CoV-2 virus into cells via angiotensin binding sites by converting enzyme-2 (ACE-2) receptors in cardiomyocytes. By binding to the ACE-2 receptor, the SARS-CoV-2 virus leads to the down-regulation of these receptors, thus reducing the synthesis of angiotensin (1–7), thereby increasing the activity of angiotensin II. The predominance of angiotensin II leads to systemic vasoconstriction, apoptosis, inflammation, and, consequently, pathological proliferation, resulting in de-novo cardiomyocyte damage or worsening of previous chronic CVD. In addition to the direct action of the SARS-CoV-2 virus, the possible mechanisms that can lead to myocardial injury and, consequently, an increase in cTn are: cytokine release, microvascular damage (due to intravascular coagulation and thrombosis), hypoxemia, and inflammation, which can destabilize the existing atherosclerotic plaque [15,23] (Figure 2). It has been shown that, within the COVID-19 infection, an increase in cTn correlates with an increase in other circulating markers of inflammation (IL6, IL-1, CRP, IL8, ferritin, and TNF-α), which is accompanied by disease progression and higher mortality [24]. Although different pathogenetic mechanisms can lead to myocardial injury, which of these mechanisms contribute the most to the development of ACS should be considered.

Although AMI Type 1 is a consequence of atherosclerotic plaque rupture and, consequently, coronary thrombosis, it has long been known that inflammation can affect the development of AMI Type 1 [12]. Increased production of cytokines, proteases, and free oxygen radicals by inflammatory cells leads to endothelial dysfunction and its injury [25,26].

The injury of the endothelium of the blood vessel and, consequently, rupture of the atherosclerotic plaque precede the coagulation cascade, which will result in the formation of a thrombus in the lumen of the blood vessel [27]. All three components of the Virchow triad (hypercoagulability, stasis, and vascular injury) are involved in the increased thrombosis in patients with COVID-19. As mentioned above, direct cell invasion by SARS-CoV-2 can lead to endothelial damage, in addition to inflammation [28]. COVID-19 also has increased levels of D-dimer, fibrinogen, coagulation factor VIII, and von Willebrand factor, suggesting a higher incidence of thrombosis in this disease, presenting as a stroke, pulmonary thromboembolism, or AMI [29]. Increased D-dimer values correlate with disease severity and mortality in the first 28 days of illness [30]. AMI Type 2 arises as a consequence of the disproportion between the availability and consumption of oxygen [31,32]. Hypoxemia resulting from a respiratory infection can lead to a mismatch between oxygen supply and demand, thus leading to AMI [33]. There is also evidence that COVID-19 patients with lower percutaneous oxygen saturation (SpO2) without oxygen therapy have a higher incidence of myocardial injury (*p* < 0.05) [34].

Possible mechanisms of AMI in COVID-19 also include coronary embolism (in 3% of cases) and atrial fibrillation (AF) with rapid ventricular response, which increases the probability of AMI by about two times [35,36]. Cases of AMI due to spontaneous coronary artery dissection and stent thrombosis, as well as emergency coronary artery bypass surgery (CABG), following the failure of percutaneous coronary intervention (PCI) in a COVID-19 patient, have been reported [37,38,39,40]. Although, so far, a small number of studies or individual cases correlate the occurrence of AMI with interventional/therapeutic procedures under COVID-19, and the actual incidence of these complications could be significantly higher. Finally, although extremely rare, individual cases of AMI have been reported in children with the development of multisystem inflammatory syndrome within COVID-19 [41,42,43,44].

## 5. Clinical Picture of ACS in COVID-19

The most common symptoms of ACS in patients with COVID-19 infection are fatigue, malaise, shortness of breath, chest pain, palpitations, and skipping beats. Cardiac complications include acute myocardial injury, myocarditis, heart failure, arrhythmias, and even sudden cardiac death [14,45,46]. The occurrence of sudden cardiac death in COVID-19 patients, without the possibility of obtaining biomarker confirmation, suggests that it was AMI Type 3 [12]. Arrhythmias are a common manifestation in patients with COVID-19 [47]. Hypoxemia, increased adrenergic stimulation, acid-base and electrolyte disorders (predominantly hypokalaemia (due to the influence of SARS-CoV-2 on the angiotensin–aldosterone system)), and hypomagnesaemia affect the occurrence of arrhythmias in patients with COVID-19 [48,49].

It has been shown that 16% of the patients hospitalized for COVID-19 infection develop atrial arrhythmias within 7 days (AF in 14.6% and atrial flutter in 3.8%) [50]. Incidences of 4.8% ventricular tachycardia/ventricular fibrillation and 5.6% pulseless electrical activity werereported among hospitalized patients with COVID-19 [51]. An increased incidence of sudden cardiac death during the COVID-19 pandemic has been reported in both hospitalized patients and outpatients with cardiac arrest [52].

## 6. Diagnostic of ACS in COVID-19

Diagnosing ACS can be a major challenge in patients with COVID-19.

Nonspecific changes can usually be registered electrocardiographically in patients with COVID-19. With various causes of myocardial injury, changes in the ECG may be minimal [53]. The most common change in the ECG of patients with COVID-19 who do not have ACS is sinus tachycardia [54]. The changes in the ECG that occur in AMI with ST elevation (STEMI) and AMI without ST elevation (NSTEMI) are the same in COVID patients as in patients who do not have this disease.

Elevated cTn values have been reported in patients with AMI in COVID-19. However, they may be associated with a variety of cardiac and noncardiac conditions, in addition to AMI. From cardiac conditions, elevated cTn values are registered in acute heart failure, acute myocarditis, arrhythmias, hypertrophic cardiomyopathy, etc. Noncardiac conditions accompanied by elevated cTn values are pulmonary embolism, chronic renal failure, sepsis, systemic hypoxemia, hypothyroidism, pheochromocytoma, and many others [55]. cTn is the main marker of myocardial injury, and it is necessary to consider its quantitative values, in relation to the presence of pre-existing heart disease, as well as other comorbidities that may affect its increase [53]. Lala A. et al. showed that the severity of the clinical picture and mortality of patients with COVID-19 correlated with cTn height; a minimal increase in cTn is associated with a poorer prognosis of COVID-19 patients [15]. Patients with elevated cTn levels often have a higher need for mechanical respiratory support and a higher mortality rate [56]. A moderate increase in cTn (less than 2–3 times ULN) does not require AMI protocol treatment, unless its increase correlates with a clear clinical picture of AMI and ECG changes [53]. In patients with moderate cTn elevations, echocardiography is recommended to assess the global and regional left ventricular function. Detailed clinical assessments, including the characteristics of chest pain, assessment of severity of COVID-19 infection, measurement of high sensitive cTn at initial presentation and after 3 h, and echocardiographic examination, are important in the algorithm for diagnosing AMI in patients with COVID-19 [53]. Patients with typical ECG changes and a significant increase in cTn require treatment according to the European Society of Cardiology (ESC) and American College of Cardiology/American Heart Association (ACA/AHA) recommendations for the treatment of AMI.

Catheterization with angiography should be performed in all patients with ACS in COVID-19, in order to diagnose coronary heart disease, as well as in patients with STEMI/NSTEMI—in therapeutic terms, the implantation of endovascular prostheses in coronary blood vessels with significant stenosis. It is increasingly recognized that there is a group of AMI patients with no angiographic obstructive CAD (MINOCA). The prevalence of MINOCA is estimated to be 6–8% among the patients diagnosed with AMI; it is more common in women than men, as well as in patients presenting with NSTEMI, compared with those presenting with STEMI [12]. In a study by Stefanini G. et al., it was shown that, in patients with COVID-19 with ST elevation and regional wall movement abnormalities, the absence of obstructive coronary lesions was present in approximately 40% of patients [57]. This presents a great difficulty in diagnosing AMI in these patients.

CT angiography, as a non-invasive method of imaging coronary blood vessels, may be considered in patients with suspected AMI and COVID-19. CT angiography is an important method for the verification of COVID-19 pneumonia, as well as the gold standard in the diagnosis of PE, which is often diagnosed in patients with COVID-19 [58,59].

Magnetic resonance imaging in COVID-19 infection is recommended in patients with suspected myocarditis and as a possible means of diagnosing AMI without coronary artery occlusion [60]. Myocardial injury in COVID-19 can be confirmed in patients after hospital treatment via magnetic resonance imaging [61].

Due to the mixed symptoms of both COVID-19 and its possible associated diseases, dilemmas in diagnosing ACS in COVID-19 are still common today.

## 7. Treatment of ACS in COVID-19

There have been clear delays in the use of STEMI treatment regimens in patients who require urgent PCI (especially within the first 90 min of AMI symptoms) during the COVID-19 pandemic [62]. A large number of STEMI centers are turning into COVID hospitals during the COVID-19 pandemic. The treatment of patients with AMI is not only compromised for STEMI patients, but also for patients with NSTEMI. According to the recommendations of the European Society of Cardiology and American Society of Cardiology, all patients with NSTEMI who have chest pain on admission, signs and symptoms of heart failure, and malignant arrhythmias should undergo emergency coronary angiography and possible PCI [63,64]. Even in patients who do not have the above acute conditions and chest pain, but have been diagnosed with NSTEMI, coronary angiography must be performed within the first 24 h of initial presentation [64]. Due to the COVID-19 pandemic, many patients with ACS were discharged from the hospital earlier than under the ACS protocol. This has resulted in more AMI complications [65]. Another reason for the occurrence of complications in these patients is the lower possibility of follow-up examinations in emergency conditions.

The predisposition of COVID-19 STEMI patients to thrombosis, on the one hand, and non-adherence to the therapeutic regimen, on the other hand, has caused a higher percentage of stent thrombosis. The results of a study by Choudry FA et al. showed that STEMI patients with COVID-19 were more prone to thrombosis than non-COVID STEMI patients [66]. The reasons for the more frequent stent thrombosis in STEMI patients are the presence of multiple culprit lesions, higher thrombus grade, lower resultant myocardial blush grade, associated increased use of GP IIb/IIIa inhibition, and thrombus aspiration [66]. The incidence of stent thrombosis in the pre-COVID era ranged from between 1–4%, while it ranged from 8.1% to 21% during COVID-19 [67]. Early stent thrombosis is accompanied by a mortality of up to 45% [68].

According to the guidelines for the treatment of NSTEMI and STEMI infarction, dual antiplatelet therapy (DAPT) is recommended, which includes acetyl salicylic acid in combination with P2Y12 inhibitors (ticagrelor or prasugrel). Regardless of the method of treatment for AMI, STEMI, and NSTEMI, patients should usually receive DAPT for one year. Prolongation of DAPT after 12 months in patients after STEMI should be considered, in case of the high risk of new ischemic events and in the absence of the risk of bleeding. With an increased risk of bleeding and lower ischemic risk, a shorter application of DAPT for 3–6 months can be considered; with a very high risk of bleeding, as long as 1 month could be considered [62,64].

The use of triple therapy in ACS is indicated in the cases of patients with AF, mechanical valvulae, and venous thromboembolism. The use of oral anticoagulant therapy should be consistent with the individual risk of bleeding, as well as the ischemic risk. In particular, the risk of bleeding should be considered more frequently in COVID-19 patients, because a large number of patients, especially those with a severe clinical picture of COVID-19, have been shown to develop thrombocytopenia [69,70]. Both the HAS-BLED (the risk of bleeding is assessed) and CHA2DS2VASc (the risk of thrombo-embolic events is assessed) scores are most commonly used for risk assessment.

According to the guiding recommendations, in case of acceptable hemorrhagic risk, triple therapy of NOACs with a P2Y12 inhibitor, as well as aspirin, is recommended for the first week to one month; after that, it is necessary to continue NOACs with a P2Y12 inhibitor, until the expiration of 12 months since ACS, after which, only the NOACs are left in therapy [71]. When interacting with NOACs, care should be taken regarding the possible interactions of the drugs used to treat the COVID-19 infection. Macrolide antibiotics could increase the serum concentrations of dabigatran, betrixaban, and edoxaban, through the inhibition of P-glycoprotein [72]. The potential drug–drug interactions between dexamethasone and methylprednisolone with apixaban and rivaroxaban have been described in the literature [73]. The concomitant use of corticosteroids and these NOACs may reduce the anticoagulant effect of NOACs, which carries a risk of thrombo-embolic events [74]. There are also studies in which the effect of dexamethasone on plasma NOACs levels has not been demonstrated [75]. The American Society of Hematology suggests that rivaroxaban and apixaban have significant drug interactions with ritonavir, a component of the antiviral PAXLOVID (nirmatrelvir (300 mg) with ritonavir (100 mg)). Co-administration will increase the concentration of apixaban or rivaroxaban and may increase the risk of bleeding [76,77].

Previous studies have suggested the possible benefit of antiplatelet therapy in some patients with COVID-19 [78]. A RECOVERY study conducted on 14,892 patients with ACS-independent COVID-19 infection showed that aspirin did not improve the clinical outcome [79]. Aspirin use was associated with a reduction in thrombotic events (4.6% vs. 5.3%) and increase in major bleeding events (1.6% vs. 1.0%). Some studies suggest that an earlier administration of antiplatelet therapy may lead to a reduction in thrombosis in COVID-19 patients [69].

Apart from the fact that COVID-19 is a prothrombogenic condition, the occurrence of heavy bleeding in these patients in intensive care units is common and estimated at about 12.4% (major bleeding 5.7% vs. non-major bleeding 6.7%) [80]. Major bleeding is defined as any potentially fatal bleeding that occurs at certain sites (e.g., intracranial, intraspinal, intraocular, retroperitoneal, intraarticular, pericardial, or intramuscular with compartment syndrome) and causes a hemoglobin drop of 2 g/dL or more, requiring requires the use of a blood derivative [81]. It can be provoked by infection, the appearance of acidosis, and the effect of applied antiplatelet and anticoagulant therapy on the already compromised gastric mucosa. Excessive bleeding occurs in 3% of patients with COVID-19 who are receiving therapeutic doses of anticoagulant therapy, 1.7% of patients receiving prophylactic doses of anticoagulant therapy, and 1.9% of patients not receiving anticoagulant therapy. The bleeding rate with COVID-19 is higher in patients receiving low molecular weight heparin than in direct oral anticoagulant drugs (2.6 vs. 1.3%, respectively) [82]. The occurrence of AF in COVID-19 patients was associated with a higher incidence of bleeding, compared to patients without AF (10.6% vs. 2%, respectively) [83,84]. Given the often-high ischemic risk, as well as the often-high risk of bleeding, and analyzing the benefit/risk ratio in these patients, the treatment of patients with AMI and COVID-19 is often associated with dilemmas and difficult decisions.

At the beginning of the COVID-19 pandemic, the use of angiotensin-converting enzyme inhibitors (ACEIs) was debatable. The SARS-CoV-2 virus binds to ACE-2 receptors via the S protein, through which it reaches the cell via endocytosis and continues to replicate. Elderly people, as well as people diagnosed with CVD, have higher ACE-2 receptor expression [85]. Although ACEI use is associated with increased ACE-2 receptor expression, studies have not shown increased mortality in patients who have previously used ACEI. A retrospective, multicenter study of 1128 adult patients with hypertension and COVID-19 infection (188 patients taking ACEI or angiotensin 2 receptor blockers (ARBs) before and during hospitalization) showed a lower risk of all causes of death in the ACEI/ARB group [86]. These results were supported by a large prospective cohort study, according to which, the use of ACEI and ARB is associated with a milder clinical course of the disease [87]. Such findings were confirmed by a meta-analysis by Wang Y et al. [88]. A joint statement by the Council on Hypertension and European Society of Cardiology has advised physicians to continue treatment with their current ACEI/ARB regimen, even though these drugs can raise ACE2 levels [89].

The importance of statin use in patients with COVID-19 and its associated CVD can be reflected in the already-known pleiotropic, anti-inflammatory, and immune-modulatory effects [90]. In addition to these effects, studies suggest the possible effect of statins on the coronavirus main protease (M pro), which plays an important role in the proteolythic maturation of the virus [91,92]. According to the data in an in silico study by Reiner Ž. et al. high statin binding energy for M pro was shown, thus indicating the possible role of statins in the treatment of COVID-19 [91]. There is also in vitro evidence of the antiviral effect of statins on certain strains of the SARS-CoV-2 virus [93]. According to a meta-analysis by Vahedian-Azimi A. et al. statin use in patients with COVID-19 showed a statistically significant reduction in mortality [94]. When using statins in patients with COVID-19, possible drug interactions should always be considered. According to the HEART UK experts, atrovastatin, simvastatin, and pravastatin should not be combined with remdesivir—they should be replaced by rosuvastatin [89]. The temporary suspension of statins is also recommended in the case of macrolide antibiotics or tocilizumab use. It is recommended that all hospitalized patients be monitored regularly for transaminases; in the case of extremely high values, especially if the statins are used in combination with remdesivir, which shows significant hepatotoxicity, statin discontinuation is advised [95].

Currently, the data are insufficient to support the recommendations for or against the use of any vitamin, mineral, herb or other botanical, fatty acid, or other dietary supplement ingredient for the prevention or treatment of COVID-19 [96]. Some authors suggest that the use of several dietary supplements, such as garlic, ginger, cranberries, oranges, omega-3 and -6 polyunsaturated fatty acids, vitamins (e.g., vitamins A, B vitamins, C, D, and E) and minerals (e.g., Cu, Fe, Mg, Mn, Na, Se, and Zn), have potential antiviral activity [97].

## 8. Impact of COVID-19 on Mortality from AMI

Many studies have shown a higher mortality of COVID-19 patients in whom myocardial injury occurred, compared to COVID patients in whom it did not develop [98,99]. The results of a meta-analysis of 13 studies by Santoso A. et al. (in 2389 patients) showed that acute myocardial injury within COVID-19 infection was associated with a more severe clinical picture (RR 13.81, *p* < 0.001; I2: 0%), more frequent hospitalizations in intensive care units (RR 7.94, P ¼ 0.01; I2: 79%), and higher mortality rates (RR 7.95, *p* < 0.001; I2: 65%) [100].

COVID-19 patients with ACS are more likely to have a more severe form of infection, and they have a mortality rate of up to 30%. The rehospitalization rate is very high (up to 20%) within the three months of follow-up [18]. The results of an observational cohort study (12,958 AMI patients) (519 COVID-19 positive) also showed that patients with COVID-19 and ACS had higher in-hospital (OR: 3.27; 95% (CI): 2.41–4.42) and thirty-day (OR: 6.53, 95% CI: 5.1–8.36) mortality, compared to patients with ACS without a diagnosis of COVID-19 [101]. The analysis of the Rodriguez-Leor O. et al. study reveals that patients with COVID-19 and AMI have a higher incidence of stent thrombosis (3.3% vs. 0.8%, *p* = 0.020) and cardiogenic shock after PCI (9.9% vs. 3.8%, *p* = 0.007). In this study, the in-hospital mortality rate was higher in the group of patients with COVID-19 and AMI, compared to those with AMI without COVID-19 (23.1% vs. 5.7%, *p* < 0.0001) [102].

## 9. The Impact of the COVID-19 Pandemic on the Outcome of Patients with AMI

Since the beginning of the pandemic, many countries have reported a 19–40% reduction in the incidence of AMI, compared to the period before COVID-19 [103,104,105]. The reluctance of patients with typical anginal problems to seek medical help, and in the desire to avoid admission to the hospital, for fear of COVID-19 infection, led to a “false” reduction in the number of hospitalizations for various diseases. Additionally, the results of a study from Italy (547 patients hospitalized for ACS—45% STEMI) showed that the average admission rate for ACS was 13.3 per day, compared to 18.9 admissions per day during the previous non-COVID year. A significant increase in mortality in these patients has also been reported [106]. A reduction in the number of AMI hospitalizations during the early period of the COVID-19 pandemic, as well as in the results of a cohort study in 15,244 patients (4955-STEMI/10. 289-NSTEMI), was demonstrated. Increased mortality of these patients was also registered, with the largest increase for patients with STEMI [103]. Patients with typical anginal symptoms within the AMI, by postponing a visit to the doctor, conditioned the extension of the time from the appearance of symptoms to the first medical contact. For this reason, the incidence of AMI complications and mortality rates increased in the pre-COVID era, compared to the COVID era. The meta-analysis analyzed 79 studies from over 57 countries. In this meta-analysis, a decrease in the number of hospitalizations, due to STEMI during the COVID-19 pandemic, was registered, in relation to the observed period before the pandemic (OR 0.80, 95% CI 0.76–0.84; *p* < 0.0001) [107]. In the COVID era, fewer patients under went PCI. Patients with ACS have a worse prognosis in the COVID era than before the COVID era [108,109]. In a study by He L. et al. the number of hospitalized patients with STEMI and NSTEMI was also reduced; however, by reducing the number of elective PCIs and opening up space for more emergency PCI procedures in critically ill patients with AMI, the mortality rate was similar in the COVID era, compared to the pre-COVID era [110].

Aldujeli A. et al. showed that, in a population of 200 STEMI patients, there was a significant delay from onset of symptoms to hospitalization in the COVID-19 era; however, the time of arrival to the hospital, i.e., the “door to balloon time” (DTBT), has not changed, in relation to the pre-COVID-19 era [111]. In contrast, Garcia S. et al. showed an average of 20% prolongation of DTBT during a pandemic [112]. By analyzing the results of a study that compared 34,127 STEMI patients undergoing primary PCI during the period 2017–2019 with patients who had PCI during the period from January to April 2020, we can see that DTBT increased from an average of 37 min (16–94 min) to an average of 48 min ((21–112 min); *p* < 0.001) [113]. With a delay in the implementation of PCI, the zone of necrotic myocardium, degree of heart failure, and risk of mortality increase.

## 10. Conclusions

Due to its pathogenesis, the COVID-19 disease is often the cause of CVD, which is why it is even more often followed by a higher mortality rate. If patients with chronic CVD receive COVID-19, they often have an acute exacerbation of chronic CVD, possible development of ACS, and worse prognosis.

With the development of the COVID-19 pandemic, the total number of ACS in the world population has increased; however, due to the fear of infection, patients rarely report to health institutions, which is why the “seemingly reduced” number of hospitalizations of these patients is registered. An increase in sudden cardiac death has been reported in the COVID era, which may also explain the lower number of hospitalizations of ACS. There are dilemmas as to whether adequate ACS statistics are possible in the COVID-19 pandemic era.

In the COVID era, a delay in the first medical contact is also registered. The COVID era is also characterized by delays in the application of therapeutic procedures, frequent occurrence of AMI complications, and higher mortality of patients with CVD, compared to the pre-COVID era.

Although the time of the hospitalization of patients with COVID-19 and ACS has been extended, in most countries, the time of the hospitalization of patients with ACS without COVID-19 has been shortened (due to overcrowding of COVID patients), which has led to more frequent AMI complications.

Due to the mixing of COVID-19 and ACS symptoms, nonspecific ECG findings, and elevated cTn values (which have also been seen in a large number of COVID-19 patients without ACS), the diagnosis of ACS in these patients is difficult and accompanied by many dilemmas.

The treatment of patients with ACS in the pre-COVID era, compared to the COVID era, is only seemingly the same; it is difficult and accompanied by various dilemmas. Patients with ACS and COVID-19 have an increased prothrombogenic risk, and they frequently have an increased risk of bleeding; therefore, deciding on the right treatment regimen is extremely difficult.

The prognosis of patients with COVID-19 is often uncertain; if COVID-19 is associated with ACS, the prognosis becomes even more uncertain. In order to resolve all dilemmas in the approach, diagnosis, and treatment of patients with COVID-19 and ACS, it may be necessary to wait for the results of future, large, multicenter studies, which would guarantee the greater survival of these patients.

## Data Availability

Not applicable.
